# Study on the uptake of Gastrodin in the liver

**DOI:** 10.1016/j.heliyon.2024.e36031

**Published:** 2024-08-08

**Authors:** Xing Wang, Wenzhu Yang, Jitong Lv, Xinya Liao

**Affiliations:** a College of Medicine, Southwest Jiaotong University, No. 111, Chengdu North 2nd Ring Road, Chengdu, Sichuan, 610003, China; b School of Life Science and Engineering, Southwest Jiaotong University, No. 111, Chengdu North 2nd Ring Road, Chengdu, Sichuan, 610003, China

**Keywords:** Gastrodin, Pharmacokinetics, Hepatic uptake, OATPs, OCTs

## Abstract

**Background:**

Gastrodin is the active monomer of the Chinese herb Rhizoma Gastrodiae with the largest quantity of active components. Gastrodin is commonly used in the treatment of central nervous system disorders such as headaches and epilepsy due to its sedating and hypnotic properties. Its pharmacological mechanism and clinical application have been extensively explored due to its low toxicity.

**Methods:**

To investigate the molecular mechanism of hepatic uptake of Gastrodin in rats, animals were randomly assigned to three groups: control group, rifampicin (RIF) group, and adrenalone (ADR) group. Blood samples were collected through the cardiac puncture 90, 180, and 300 min after injection, respectively. Rats were sacrificed 300 min after administration, and liver tissue was collected. Gastrodin concentration was determined by HPLC, and the Kp value was calculated.

**Results:**

After administering the inhibitors of organic cation transporters (OCTs) and organic anion transporting polypeptides (OATPs), the K_P_ values in the experimental groups were significantly lower compared to the blank control group (P < 0.05).

**Conclusions:**

These findings imply that Gastrodin may be a substrate for both OCTs and OATPs.

## 1 Introduction

**Fig. 1:**
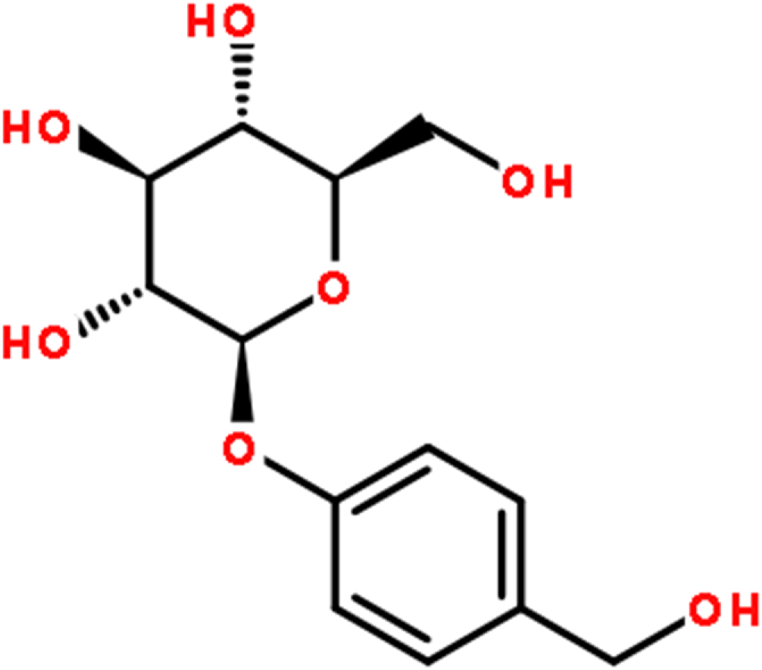
The chemical structure of Gastrodin.

Gastrodia elata is a Chinese traditional medicine, the highest effective monomer and active ingredient content in the chemical called for hydroxyl methyl benzene - beta - D - pyran glucoside, also known as Gastrodin (The chemical structure is shown in Fig. 1). It is a kind of phenolic glycoside, chemical formula for C_13_H_18_O_7_, relative molecular weight is 286 Da, are the main bioactive ingredients Gaston dragon [1,2]. In recent years, researchers have made a lot of exploration and research on the clinical application of Gastrodin, and found that Gastrodin has sedative and hypnotic properties, analgesia, protection of nerve cells and brain damage cells. For example, Gastrodin has been shown to ameliorate renal hypertension in rats by inhibiting autophagy signaling [3]. Recent study indicates that Gastrodin could prolong the lifespan by regulating the antioxidant ability, and protect against neurodegeneration in the Pink1B9 model of PD. This suggests that Gastrodin can be considered as an ideal therapeutic candidate for drug development towards anti-aging [4].

Drug pharmacokinetic including drug absorption, distribution, metabolism and excretion, these processes are dependent on the drug transporters. Among them, organic cation transporters (OCTs) in the absorption, distribution and excretion of the substrate plays an important role in the process of, including the liver, kidney and gastrointestinal tract in the role of organic cation transporter most obvious [5]. Organic anion transfer peptides (OATPs) can transport all kinds of structure of different drugs, and in the control of drugs and their metabolites in the process of the pharmacokinetic has extremely important significance. OATPs are expressed in tissues such as liver, kidney, and small intestine. Meanwhile, OATPs can transport a wide range of substrates. Not only can it participate in the transport of organic anions, but it can also participate in the transport of certain organic cations [6,7].

The transporters involved in liver transport mainly include organic anion transport peptides (Oatps) and organic cation transporters (Octs), and these two transporters are most expressed in the liver. Therefore, this study chose Oatps and Octs as the research objects. Transport proteins affect drug efficacy and toxicity, the liver is the main organ for the metabolism of substances [8]. Previous pharmacokinetic studies in our laboratory on different doses of Gastrodin revealed that the pharmacokinetic profile of Gastrodin is non-linear in mice and conforms to a two-compartment model [9], preliminary judgment that transporters involved in Gastrodine uptake in the liver. Using the method of rats in vivo, and choose organic anion transfer peptide Oatps and organic cation transporter Octs inhibitors for the probe, after the transporters are suppressed, intravenous drip Gastrodine, drug concentrations reach homeostasis in blood drug concentration, drug absorption rate and elimination rate balance, illustrate the liver intake transporters saturated. Using HPLC determination of drug concentration in liver and blood at this time the ratio of the Kp (Kp = Css/Css drug concentration of liver blood drug concentration), and under normal circumstances (i.e., without giving inhibitors) compared to Kp, if Kp decrescent, namely that the drug transporters of the substrate. This study explore the intrinsic relationship between the uptake of Gastrodin in rat liver and the organic anion transport peptide Oatps and organic cation transporter Octs, thus preliminarily elucidating the molecular mechanism of Gastrodin uptake in the liver, provide theoretical basis for the safety of clinical combination therapy [[10], [11], [12]].

## 2 Methods

### 2.1 Animals and grouping

The use of experimental animals was approved by the Ethics Committee of Southwest University (Chongqing, China). We strictly followed the National and Institutional guidelines for animal protection and welfare. In total, 18 adult male Sprague-Dawley (SD) rats (SPF, weight: 150 ± 20g) were obtained from the Center of Experimental Animals, Sichuan Province People's Hospital. The rats were kept at room temperature (25 °C) with free access to food and water. They were allowed to adapt to the new environment for one week before starting induction and treatment. Following acclimatization, the experimental rats were randomly divided into three groups (n = 6) as follows: Control group (treated with Gastrodin), ADR group (treated with Gastrodin and adrenalone), and RIF group (treated with Gastrodin and rifampicin).

### 2.2 Chemicals and reagents

The chemicals and reagents used in this study included a Gastrodin extract (Xi'an Baichuan Biotechnology Co., Ltd., GAS standard (China Institute of Food and Drug test), adrenalone, rifampicin (Shanghai Merrill Chemical Technology Co., Ltd.), phloroglucinol (China Pharmaceutical Shanghai Chemical Reagent Co., Ltd.). All other reagents were chromatographically graded.

### 2.3 Chemical reagent preparation

#### 2.3.1 Standard solutions

To clarify the role of hepatic transport proteins, the concentration of Gastrodin chosen for this experiment was 569 mg kg^−1^ (converted from the concentration in mice: rat dose (mg·kg^−1^) = mouse dose (mg·kg^−1^)/1.44), based on the results of previous studies [8]. An equal dose of Gastrodin was injected before intravenous drip as a loading dose, to ensure that the steady-state blood concentration (C_ss_) of Gastrodin was reached and the transporter protein was saturated by 300 min of intravenous drip. Then, the volume of Gastrodin was computed according to the following formula: drop volume = drop speed (4 μL h^−1^ g^−1^) × time × weight.

Gastrodin solution: 1138.9 mg of Gastrodin extract was added to a 10 mL volumetric flask. Physiological saline was added and diluted to prepare a solution with a concentration of 113.89 mg mL^−1^. Then, the solution was frozen and stored (administered at 1 mL·200g ^−1^ i.v.).

Adrenalone (ADR) solution: 64 mg of adrenaline was weighed precisely and prepared at 8 mg mL^−1^ concentration (administered at 0.5 mL·200 g^−1^ i.v.) using 30 % PEG400 as solvent.

Rifampicin (RIF) solution: 90 mg of rifampicin was precisely weighed. Then, 30 mL saline was added to prepare 3 mg mL^−1^ solution (administered at 2 mL·200 g ^−1^ i.g.) [13].

#### 2.3.2 Stock solutions

Reference solution: reference solutions were prepared at a concentration of 1 mg mL^−1^ using 10 mg Gastrodin containing moderate methanol volumetric flask.

Internal standard (IS) solution: 20 mg of phloroglucinol was weighed and added to moderate amounts of methanol to produce 2 mg mL^−1^ standard solution.

All solutions were stored at 4 °C.

### 2.4 Administration regimen

The rats were fasted for 12 h before the experiment. They had free access to water. The rats were regularly weighed before the initiation of the experiment. The control group first received Gastrodin solution (569 mg kg^−1^) through the caudal vein as a loading dose, followed by the caudal injection of a Gastrodin solution (569 mg kg^−1^) at a rate of 4 μg h^−1^·g^−1^. Each rat of the RIF group was given rifampicin 2 mL·200 g^−1^ via oral gavage and 1h later 569 mg kg^−1^ of Gastrodin was injected [14]. Likewise, the ADR group was injected with adrenalone (0.5 mL·200 g^−1^) via the caudal vein, and 0.5 h later 569 mg kg^−1^ of Gastrodin was injected. Blood samples were collected 90, 180, and 300 min after administration and stored in a sodium heparin-containing tube, shaken up and down to fully mix with sodium heparin and avoid coagulation, and stored at −20 °C. The rats were treated by intraperitoneal injection of pentobarbital sodium for 300 min, and their livers were quickly harvested, washed three times with normal saline, and dried with filter paper.

### 2.5 Sample preparation

#### 2.5.1 Blood samples

First, 100 μL blood sample was mixed with 10 μL of standard solution. The mixed solution was centrifuged at 3500 r·min^−1^ for 10 min after adding 400 μL methanol. Then, the supernatant was aspirated and blow-dried with nitrogen in a water bath at 37 °C. To obtain the target supernatant, it was then lysed in double-distilled water and centrifuged at 10000 r·min^−1^ for 10 min.

#### 2.5.2 Liver tissue

Liver tissue was put in an appropriate amount of saline and tissue homogenate was prepared. Then, the solution was centrifuged at 3000 r·min^−1^ for 10 min and the supernatant was extracted. Next, 100 μL of tissue homogenate and 10 μL of IS solution were added. Then, 400 μL of methanol was added, mixed and centrifuged for 10 min and the entire supernatant was aspirated [15]. Next, the sample was blow-dried with nitrogen in a 37 °C water bath, re-dissolved in 200 μL of double-distilled water, vortexed, mixed, and centrifuged at 10,000 r·min^−1^ for 10 min. The target supernatant was aspirated once more.

#### 2.5.3 Chromatographic conditions

An Agilent 1260 high-performance liquid chromatography spectrometer (HPLC, Agilent Technology Co., USA) was used for the identification of chemical compounds in Gastrodin extract, rat plasma, and liver tissue. The chromatographic separation was conducted at 30 °C on a Waters™ C_18_ column (4.6 × 250 mm, 5.0 μm). The mobile phase was acetonitrile/0.05 % phosphoric acid (3:97, V/V) with the following gradient elution. The sample volume was 10 μL. The detection wavelength was set to 220 nm.

### 2.6 Calibration standards

The calibration standards were prepared by spiking 10 μL of IS solutions and adequate reference solution into 100 μL of blank rat plasma or blank rat liver tissue to obtain the final concentrations of 0.1 μg mL^−1^ -500 μg mL^−1^. The sample was processed as described above, then a 10 μL aliquot was added to the HPLC system for analysis.

### 2.7 Methodological validation

We aimed to confirm the selectivity of this protocol by respectively adding a blank plasma sample, a reference solution, and a standard solution during the experiment. At the same time, the specificity was assessed by comparing the retention times of the analyses in different solutions.

#### 2.7.1 Selectivity and specificity

Blank blood samples were spiked with Gastrodin (1000 μg mL^−1^) and the IS solution (2000 μg mL^−1^) and subjected to HPLC under defined chromatographic conditions. The blank blood sample was then measured under the same chromatographic conditions and the graphs of the two chromatographic results were compared. The specificity of the liver tissue was validated in the same manner.

#### 2.7.2 Accuracy and recovery

Parallel aspirations of 100 μL of blank rat plasma or blank rat liver homogenate were added to the appropriate amount of Gastrodin stock solution to prepare sample solutions (after extraction) at concentrations of 50 μg mL^−1^, 100 μg mL^−1^, 500 μg mL^−1^. In addition, 50 μg mL^−1^, 100 μg mL^−1^,500 μg mL^−1^ unextracted and treated standard blood sample solutions were prepared and measured.

#### 2.7.3 Stability

To verify whether the sample processing and detection methods were stable, we examined both their room temperature stability and their repeated freeze-thaw stability.

Three solutions containing 50 mg mL^−1^, 100 mg mL^1,^ and 500 mg mL^−1^ of Gastrodin were first prepared and stored at room temperature. This was then measured every 6 h. Finally, the RSD was calculated, which was examined for its room temperature stability.

Three solutions of the same concentration were prepared as described above and tested immediately after preparation. They were then stored at −20 °C for 21 h, removed to allow thawing, and left at room temperature for 3 h before assay. The test was continued at a low temperature and the RSD was calculated after repeating the assay three times to investigate its repeated freeze-thaw stability.

#### 2.7.4 Precision

First, 100 μL of plasma or liver homogenate from blank rats was aspirated and prepared at concentrations of 50, 100, and 500 mg mL^−1^ of Gastrodin and measured on the same day and four consecutive days to calculate the intra-day precision and inter-day accuracy.

#### 2.7.5 Reproducibility

First, 100 μL of a blank blood sample or blank rat liver homogenate was precisely aspirated. Next, an appropriate amount of Gastrodin stock solution was added to prepare a sample solution with 100 μg mL^−1^ concentration of Gastrodin. The sample was injected 6 times and the RSD was calculated.

## 3 Results

### 3.1 Method validation

#### 3.1.1 Selectivity and specificity

**Fig. 2:**
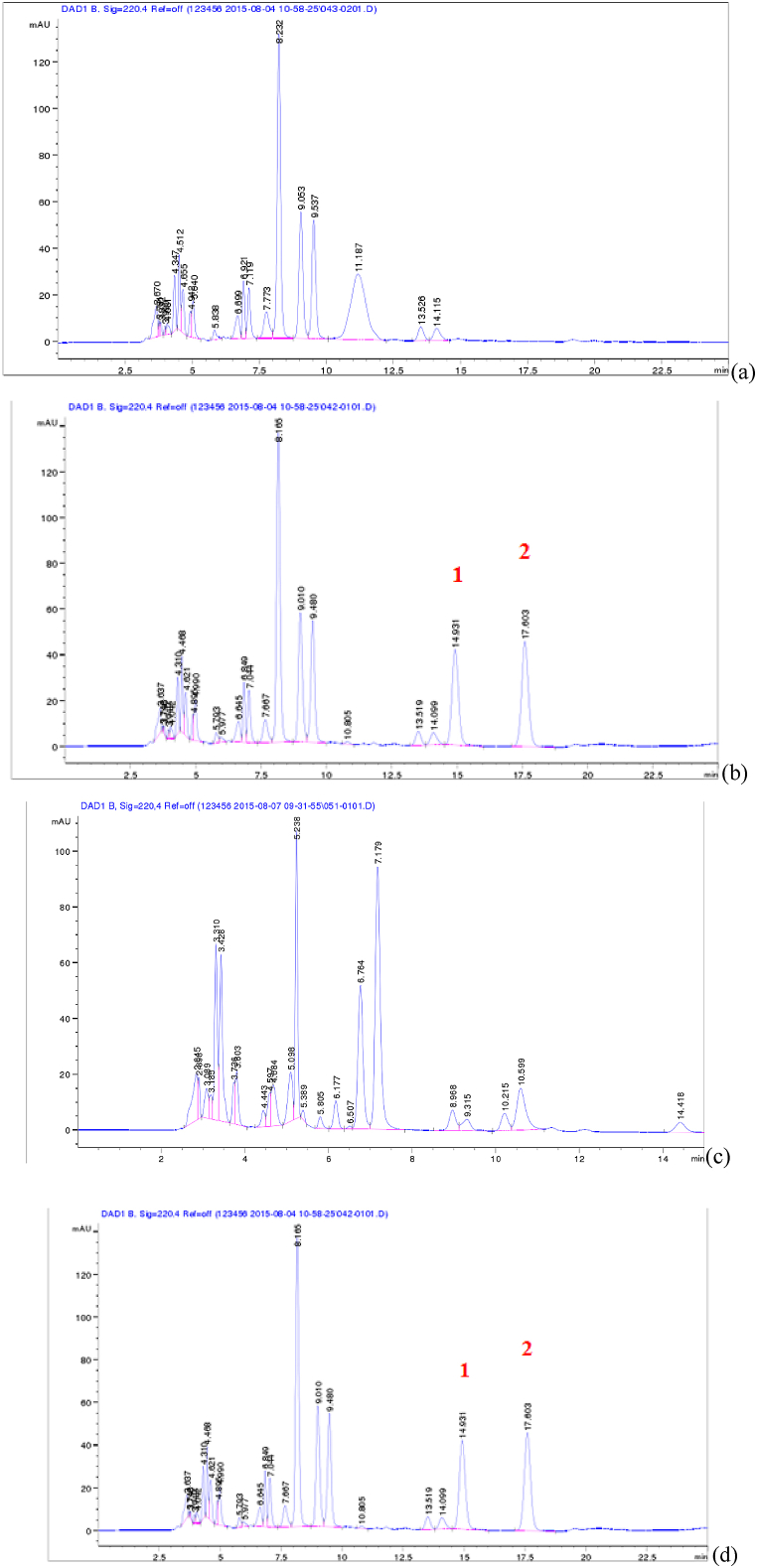
Validation of the method in terms of selectivity and specificity. (a) The untreated chromatograms of plasma samples from rats without any treatment. (b) The chromatograms of GAS (1) and phloroglucinol (2) blank samples. (c) The chromatograms of liver tissue from rats that were not given any treatment. (d) Liver tissue chromatograms tissue in rats injected with GAS (1) and phloroglucinol (2).

As shown in Fig. 2(a) and (b), the peaks of Gastrodin and phloroglucinol were completely different from the peaks of endogenous protein impurities in blood samples, without mutual interference. Similarly, as shown in Fig. 2(c) and (d), the chromatographic peaks of Gastrodin and phloroglucinol were completely different from the chromatographic peaks of endogenous protein impurities in liver tissues, without mutual interference.

#### 3.1.2 Accuracy and recovery

As shown in Table 1, the results showed that the extraction recoveries were all above 80 % in blood samples and above 75 % in liver tissue homogenates. The accuracy of Gastrodin was good, indicating that the method was stable.

**Table 1:** Accuracy and extraction recovery of blood liver samples of GAS.

Analyte	Concentration (μg·mL^−1^)	Not extracted samples (μg·mL^−1^)	Extracted samples (μg·mL^−1^)	Accuracy (%)	Extraction recovery (%)
Plasma	50	46.8	38.3	93.6	81.8
	100	89.2	76.7	89.2	86.0
	500	481.3	342	96.2	96.2
Liver	50	47.3	36.3	94.6	76.9
	100	87.9	82.9	87.9	94.3
	500	479.5	453.5	95.9	94.7

#### 3.1.3 Stability

The results showed that the relative standard deviation of the Gastrodin solution was less than 10 % at freeze-thaw cycles, low temperature, and room temperature, indicating that the Gastrodin solution can be stored at −20 °C for at least 3 days with good stability.

#### 3.1.4 Precision

Inter-day and intra-day precision showed that both blood samples and liver tissues met the standards for biological samples (Table 2).

**Table 2:** Intra-day and inter-day precision of blood and liver samples.

Analyte	Concentration (μg·mL^−1^)	Inter-day RSD (%)	Intra-day RSD (%)
Plasma	50	3.14	3.07
	100	5.19	1.92
	500	3.14	6.25
Liver	50	7.94	5.21
	100	5.89	8.65
	500	2.78	2.72

#### 3.1.5 Reproducibility

The RSD was calculated after six repetitions of the sample solution. The RSD of Gastrodin was 1.99 % in liver tissue homogenates, indicating that the experiment was repeatable.

### 3.2 Drug concentration in rat plasma and liver tissue

As shown in Table 3, the concentrations of Gastrodin in plasma and liver tissues were collected 90, 180, and 300 min after giving the medication. The result shown that the Kp value of Control group is larger than that of ADR group and RIF group. In order to further compare whether there is a significant difference, SPSS statistics17.0 software was used for analysis. After independent sample *t*-test, the Kp values of Control group and ADR group showed a significant difference with P = 0.033 < 0.05; The Kp values of Control group and RIF group were tested using independent samples *t*-test, and P = 0.012 < 0.05, indicating a significant difference. So, it can be seen that the Kp value of Control group is significantly higher than that of ADR group and RIF group.

**Table 3:** Concentrations of GAS in liver and blood samples in each group.

Group	Plasma(μg·mL^−1^)			Liver(μg·mL^−1^)	K_P_	K_P_ Value
	90min	180min	300min			
**Control -1**	418.6414	296.4502	160.2300	46.7539	0.2918	0.1401 ± 0.1269
**Control-2**	490.4070	450.2900	435.3740	94.5472	0.2172	
**Control -3**	858.0713	241.1238	690.0752	42.6760	0.0618	
**Control -4**	839.0300	473.1890	482.2634	154.5100	0.3204	
**Control -5**	374.1060	388.0036	537.2563	49.3480	0.0919	
**Control -6**	714.8180	943.8820	961.3480	165.2782	0.0178	
**RIF -1**	30828.4900	13840.5600	2063.3640	143.4890	0.0695	0.0350 ± 0.0508*
**RIF-2**	1684.7620	8435.3490	443.6390	54.8940	0.1237	
**RIF-3**	8505.0480	24589.4100	8969.1250	33.3675	0.0037	
**RIF-4**	15559.1500	8312.9910	10985.5800	48.7120	0.0044	
**RIF-5**	79163.4800	20131.4400	9971.0380	41.0071	0.0041	
**RIF-6**	26044.6400	4501.0340	23912.4400	101.5434	0.0042	
**ADR-1**	762.4848	444.9557	394.4123	25.4541	0.0645	0.0691 ± 0.0444*
**ADR-2**	617.7201	650.1415	2255.8310	73.1891	0.0324	
**ADR-3**	10576.3600	3246.9130	2303.3090	106.5988	0.0463	
**ADR-4**	1294.1160	497.0489	659.0208	47.2096	0.0716	
**ADR-5**	1093.5800	1215.3470	643.2905	28.7519	0.0447	
**ADR-6**	3877.7610	1883.7120	1871.4590	290.1262	0.1550	

Compared with Control group, *p < 0.05, **p < 0.01.

## 4 Discussion

This study established an in vivo experimental method using inhibitors of OATPs and OCTs to block both transporters in rats, followed by intravenous administration of Gastrodin. After the intravenous Gastrodin had reached steady-state blood concentrations, its concentrations in the liver and blood (Kp value) were then compared. Table 3 shows that the Kp values were lower in both ADR and RIF groups compared to the control group (p < 0.05). The control group had a significantly higher Kp value than the ADR group. The Kp value refers to the ratio of drug concentration in the liver to drug concentration in the blood when the drug reaches the steady-state plasma concentration. Compared to the control group, the ADR group had decreased concentration of Gastrodin in liver tissue and increased concentration of Gastrodin in blood after the administration of OCTs inhibitors. This reflects that Gastrodin transportation from blood to the liver was inhibited after the administration of OCTs inhibitors, which implies that Gastrodin transportation to the liver may need OCTs. Similarly, Kp values were significantly lower in the RIF group than in the Control group, suggesting that hepatic transport of GAS is associated with OATPs.

As hepatic transportation mainly relies on OATPs and OCTs, and the pharmacokinetic profile of Gastrodin is non-linear, OCTs and OATPs are jointly involved in the hepatic uptake of Gastrodin. Although this in vivo study generally explored the molecular mechanism of Gastrodin transport in the liver, the cellular mechanism is yet to be uncovered.

In addition, K_P__OATPs inhibitor_ < K_P__OCTs inhibitor_ < K_P__control_, tentatively indicating that Gastrodin is a substrate for OCTs and OATPs. Selecting suitable substrates and inhibitors of OATPs and OCTs is crucial for the experiment. Therefore, our team also used OATPs and OCTs inhibitors. Based on the relevant literature, metformin hydrochloride was selected as the substrate of OCTs [16,17], and sodium sulfobromophthalide (BSP) was selected as the substrate of OATPs [18]. Although several inhibitors of OATPs and OCTs have been previously reported, there are few reports on the doses and methods of administration in animal studies. Most of the previous studies used RIF as an inhibitor of OATPs and epinephrine as an inhibitor of OCTs [19,20]. We detected drug concentration in blood samples and in the liver of rats. The concentration of metformin hydrochloride was detected by HPLC, and BSP was measured by enzyme-linked immunosorbent assay (ELISA). OCTs inhibitors resulted in a significant reduction in hepatic concentrations of the drug i.e., hepatic transportation of metformin hydrochloride was blocked by an OCTs inhibitor. Thus, ADR inhibited the hepatic transportation of OCTs in rats at a dose of 20 mg kg^−1^. RIF inhibited the hepatic transport of OATPs in rats at a dose of 30 mg kg^−1^.

Gastrodin is well known for its antiepileptic and antioxidant effects on the central nervous system [21]. In addition, studies have shown that it has preventive and therapeutic effects on liver diseases, such as fatty liver disease and several types of chronic liver disease [22,23]. In vivo and in vitro studies indicated that Gastrodin significantly relieves liver inflammation and oxidative stress by changing the composition and abundance of intestinal flora [24]. Moreover, the liver is the main organ responsible for drug metabolism, and the uptake and efflux of drugs and their metabolites are regulated by hepatic transporters. Drugs may damage transporters during liver metabolism, leading to hepatic dysfunction. Hepatic transporter proteins of one drug may be inhibited by other drugs or their metabolites [25,26]. All these challenges need in-depth studies.

## 5 Conclusions

In conclusion, the results indicated that hepatic uptake of Gastrodin relies on OATPs and OCTs. We also found that K_P__OATPs inhibitor_ < K_P__OCTs inhibitor_ < K_P__control_, tentatively suggesting that Gastrodin is a likely substrate for both OCTs and OATPs.

## Ethics statements and consent to participate

The use of experimental animals was approved by the department ethical committee of Southwest Jiaotong University (Sichuan, China), and the National and Institutional guidelines for animal protection and welfare were followed strictly.

## Consent for publication

Not applicable.

## Funding

This work was supported by the 10.13039/501100012226Fundamental Research Funds for the Central Universities (Project No.: 2682022ZTPY025).

## Data availability statement

The authors confirm that the data supporting the findings of this study are available within the article or its supplementary materials.

## CRediT authorship contribution statement

**Xing Wang:** Writing – original draft, Resources, Methodology, Data curation, Conceptualization. **Wenzhu Yang:** Writing – review & editing, Validation, Data curation. **Jitong Lv:** Writing – review & editing, Validation, Investigation. **Xinya Liao:** Writing – review & editing, Validation.

## Declaration of competing interest

The authors declare that they have no known competing financial interests or personal relationships that could have appeared to influence the work reported in this paper.
